# Supplementary material for: His unemployment, her response, and the moderating role of welfare policies in European countries. Results from a preregistered study
Source: PLoS One. 2024 Aug 20;19(8):e0306964. doi: 10.1371/journal.pone.0306964 (PMC11335131; doi:10.1371/journal.pone.0306964)
Supplement: S3 Table — (DOCX) [file pone.0306964.s003.docx]

**S3 Table. Overview of countries included in the samples**

| Country | 2009 | 2010 | 2011 | 2012 | 2013 | 2014 | 2015 | 2016 | 2017 | 2018 | 2019 |
| --- | --- | --- | --- | --- | --- | --- | --- | --- | --- | --- | --- |
| AT | X | X | X | X | X | X | X | X | X | X | X |
| BE | X | X | X | X | X | X | X | X | X | X | X |
| BG | X | X | X | X | X | X | X | X | X | X | X |
| CY | X | X | X | X | X | X | X | X | X | X | X |
| CZ | X | X | X | 0 | X | X | X | X | X | X | X |
| DE | 0 | 0 | 0 | 0 | 0 | 0 | X | X | X | 0 | 0 |
| DK | 0 | 0 | 0 | 0 | 0 | 0 | X | X | X | X | 0 |
| EE | X | X | X | X | X | X | X | X | X | X | X |
| ES | X | X | X | X | X | X | X | X | X | X | X |
| FR | X | X | X | X | X | X | X | X | X | X | X |
| GR | X | X | X | X | X | X | X | X | X | X | X |
| HR | 0 | 0 | X | X | X | X | X | X | X | X | X |
| HU | X | X | X | X | X | X | X | X | X | X | X |
| IE | X | X | X | X | X | X | X | X | X | X | X |
| IT | X | X | X | X | X | X | X | X | X | X | 0 |
| LT | X | X | X | X | X | X | X | X | X | X | X |
| LU | 0 | 0 | 0 | 0 | 0 | 0 | 0 | 0 | 0 | 0 | 0 |
| LV | X | X | X | X | X | X | X | X | X | X | 0 |
| MT | X | X | X | X | X | X | X | X | X | X | X |
| PL | X | X | X | X | X | X | X | X | X | X | X |
| PT | X | X | X | X | X | X | X | X | X | X | X |
| RO | X | X | X | X | X | X | X | X | X | X | X |
| SI | X | X | X | X | X | X | X | X | X | X | X |
| SK | X | X | X | X | X | X | X | X | X | X | X |
| UK | X | X | X | X | X | X | X | X | X | X | 0 |

Notes: “X” denotes that a country is included in the analysis and “0” denotes that a country is not included in the sample. The structure of country-years covered in the analysis is determined by the EU-SILC monthly data availability. Additionally, we dropped observations for Croatia for the years 2009 and 2010, as EUROMOD includes Croatia from 2011 onwards.
