# Supplementary material for: His unemployment, her response, and the moderating role of welfare policies in European countries. Results from a preregistered study
Source: PLoS One. 2024 Aug 20;19(8):e0306964. doi: 10.1371/journal.pone.0306964 (PMC11335131; doi:10.1371/journal.pone.0306964)
Supplement: S4 Table — (DOCX) [file pone.0306964.s004.docx]

**S4 Table. Sample descriptive statistics**

| **Explanatory variables** | **Sample A** | | **Sample B** | |
| --- | --- | --- | --- | --- |
|  | **Mean** | **SD** | **Mean** | **SD** |
| **Household characteristics** | | | | |
| Married | 0.906 | 0.292 | 0.877 | 0.329 |
| Number of children | 1.140 | 1.127 | 1.144 | 1.022 |
| Children aged 0 to 3 | 0.193 | 0.394 | 0.125 | 0.331 |
| Children aged 4 to 6 | 0.188 | 0.391 | 0.177 | 0.382 |
| Children aged 7 to 12 | 0.292 | 0.455 | 0.330 | 0.470 |
| Income |  |  |  |  |
| Quintile 1 | 0.130 | 0.336 | 0.051 | 0.220 |
| Quintile 2 | 0.234 | 0.423 | 0.119 | 0.324 |
| Quintile 3 | 0.257 | 0.437 | 0.230 | 0.421 |
| Quintile 4 | 0.212 | 0.409 | 0.300 | 0.458 |
| Quintile 5 | 0.167 | 0.373 | 0.300 | 0.458 |
| **Female partner** | | | | |
| Age | 43.073 | 9.233 | 44.009 | 8.313 |
| Education |  |  |  |  |
| Low | 0.346 | 0.476 | 0.205 | 0.404 |
| Medium | 0.469 | 0.499 | 0.464 | 0.499 |
| High | 0.185 | 0.388 | 0.332 | 0.471 |
| Occupation |  |  |  |  |
| Blue-collar low | - | - | 0.132 | 0.339 |
| Blue-collar high | - | - | 0.125 | 0.330 |
| White-collar low | - | - | 0.390 | 0.488 |
| White-collar high | - | - | 0.354 | 0.478 |
| **Male partner** | | | | |
| Age | 45.999 | 9.134 | 46.240 | 8.392 |
| Education |  |  |  |  |
| Low | 0.314 | 0.464 | 0.215 | 0.411 |
| Medium | 0.485 | 0.500 | 0.464 | 0.499 |
| High | 0.202 | 0.401 | 0.322 | 0.467 |
| Occupation |  |  |  |  |
| Blue-collar low | 0.172 | 0.377 | 0.124 | 0.330 |
| Blue-collar high | 0.388 | 0.487 | 0.303 | 0.459 |
| White-collar low | 0.145 | 0.352 | 0.133 | 0.340 |
| White-collar high | 0.295 | 0.456 | 0.440 | 0.496 |
| N couple-months | 1.192.721 | | 544.257 | |
| N couples | 36.601 | | 16.931 | |

Note: Blue-collar low skilled(ISCO 8-9), Blue-collar high skilled (ISCO 6-7), White-collar low skilled (ISCO 4-5), White-collar high skilled (ISCO 1-3)
