# Supplementary material for: His unemployment, her response, and the moderating role of welfare policies in European countries. Results from a preregistered study
Source: PLoS One. 2024 Aug 20;19(8):e0306964. doi: 10.1371/journal.pone.0306964 (PMC11335131; doi:10.1371/journal.pone.0306964)
Supplement: S5 Table — (DOCX) [file pone.0306964.s005.docx]

**S3 Table. Variance Inflation Factor analysis**

| **Explanatory variables** | **Policy variables included as control variables** | | | **Policy variables not included** | | |  |
| --- | --- | --- | --- | --- | --- | --- | --- |
|  | **Sample A** | | **Sample B** | **Sample A** | | **Sample B** | |
| Men’s unemployment | 4.67 | 4.74 | 6.08 | 1.02 | 1.01 | |  |
|  | **Household/Couple characteristics** | | |  |  | |  |
| Married *(ref. cohabiting)* | 1.10 | 1.10 | 1.11 | 1.09 | 1.09 | |  |
| Number of children | 2.74 | 2.74 | 2.40 | 2.73 | 2.39 | |  |
| Child aged 0 to 3 | 1.63 | 1.62 | 1.43 | 1.62 | 1.43 | |  |
| Child aged 4 to 6 | 1.44 | 1.44 | 1.37 | 1.44 | 1.36 | |  |
| Child aged 7 to 12 | 1.97 | 1.97 | 1.92 | 1.97 | 1.92 | |  |
| Income (*ref. Quintile 1*) |  |  |  |  |  | |  |
| Quintile 2 | 2.17 | 2.17 | 3.07 | 2.17 | 3.06 | |  |
| Quintile 3 | 2.30 | 2.30 | 4.54 | 2.29 | 4.53 | |  |
| Quintile 4 | 2.21 | 2.21 | 5.35 | 2.21 | 5.32 | |  |
| Quintile 5 | 2.16 | 2.16 | 5.66 | 2.15 | 5.62 | |  |
|  | **Female characteristics** | | |  |  | |  |
| Age | 5.30 | 5.30 | 4.99 | 5.28 | 4.97 | |  |
| Education *(ref. low)* |  |  |  |  |  | |  |
| Medium | 1.71 | 1.71 | 2.28 | 1.66 | 2.24 | |  |
| High | 1.97 | 1.97 | 3.13 | 1.94 | 3.12 | |  |
| Occupation (*ref. Blue low skilled*) |  |  |  |  |  | |  |
| Blue-collar high skilled | - |  | 1.91 | - | 1.90 | |  |
| White-collar low skilled | - |  | 2.80 | - | 2.77 | |  |
| White-collar high skilled | - |  | 3.51 | - | 3.49 | |  |
|  | **Male characteristics** | | |  |  | |  |
| Age | 4.98 | 4.98 | 4.72 | 4.97 | 4.71 | |  |
| Education (*ref. low*) |  |  |  |  |  | |  |
| Medium | 1.83 | 1.83 | 2.14 | 1.73 | 2.09 | |  |
| High | 2.35 | 2.35 | 2.94 | 2.33 | 2.91 | |  |
| Occupation (*ref. Blue low skilled* ) |  |  |  |  |  | |  |
| Blue-collar high skilled | 2.03 | 2.03 | 2.59 | 2.01 | 2.57 | |  |
| White-collar low skilled | 1.64 | 1.64 | 1.91 | 1.61 | 1.90 | |  |
| White-collar high skilled | 2.52 | 2.52 | 3.40 | 2.46 | 3.38 | |  |
|  | **Country control variables** | | |  |  | |  |
| Unemployment rate | 2.01 | 1.98 | 2.08 | 1.38 | 1.51 | |  |
| Female employment | 1.63 | 1.63 | 1.53 | 1.43 | 1.50 | |  |
| Women gender role attitudes | 3.13 | 3.14 | 2.73 | 2.86 | 2.48 | |  |
| Men gender role attitudes | 2.96 | 2.98 | 2.93 | 2.81 | 2.43 | |  |
| Childcare 0-3 (general) | 1.37 | 1.36 | 1.70 | - | - | |  |
| Childcare 4-6 (general) | 2.05 | 2.04 | 1.78 | - | - | |  |
| Childcare 7-12 (general) | 1.92 | 1.93 | 1.85 | - | - | |  |
| NRR | 4.61 | 4.64 | 6.01 | - | - | |  |
| MTR NW - PT | 1.14 |  |  | - | - | |  |
| MTR NW - FT | - | 1.16 |  | - | - | |  |
| MTR PT - FT | - |  | 1.44 | - | - | |  |
