# Supplementary material for: His unemployment, her response, and the moderating role of welfare policies in European countries. Results from a preregistered study
Source: PLoS One. 2024 Aug 20;19(8):e0306964. doi: 10.1371/journal.pone.0306964 (PMC11335131; doi:10.1371/journal.pone.0306964)
Supplement: S1 Appendix — (DOCX) [file pone.0306964.s006.docx]

**S1. Appendix. Results for models restricted to unemployment spells lasting between 3 and 6 months**

**S1.1. Labour supply increase of women (unemployment spells 3 to 6 months)**

| **Explanatory variables** | **Sample A** | **Sample B** |
| --- | --- | --- |
| Unemployment | 0.0313***  (0.0083) | 0.0360  (0.0231) |
| **Household characteristics** |  |  |
| Married *(ref. cohabiting)* | -0.0365***  (0.0101) | -0.0097*  (0.0039) |
| Number of children | -0.0129***  (0.0036) | -0.0003  (0.0028) |
| Child aged 0 to 3 | -0.0631**  (0.0187) | -0.0343*  (0.0142) |
| Child aged 4 to 6 | 0.0305*  (0.0120) | -0.0095  (0.0080) |
| Child aged 7 to 12 | 0.0149  (0.0078) | -0.0123**  (0.0050) |
| Income (*ref. Quintile 1*) |  |  |
| Quintile 2 | 0.0199***  (0.0059) | -0.0059  (0.0085) |
| Quintile 3 | 0.0548***  (0.0088) | -0.0007  (0.0095) |
| Quintile 4 | 0.0811***  (0.0115) | 0.0174  (0.0110) |
| Quintile 5 | 0.0885***  (0.0109) | 0.0282*  (0.0111) |
| **Female characteristics** |  |  |
| Age | -0.0030***  (0.0005) | -0.0028***  (0.0005) |
| Education *(ref. low)* |  |  |
| Medium | 0.0357***  (0.0082) | 0.0081  (0.0070) |
| High | 0.104***  (0.0115) | 0.0285**  (0.0088) |
| Occupation (*ref. Blue low*) | - |  |
| Blue-collar high |  | 0.0216  (0.0111) |
| White-collar low |  | 0.0210  (0.0110) |
| White-collar high |  | 0.0503***  (0.0144) |
| **Male characteristics** |  |  |
| Age | -0.0024***  (0.0004) | -0.0008  (0.0006) |
| Education (*ref. low*) |  |  |
| Medium | 0.0060  (0.0059) | -0.0087  (0.0084) |
| High | -0.0079  (0.0054) | -0.0133*  (0.0066) |
| Occupation (*ref. Blue low*) |  |  |
| Blue-collar high | -0.0051  (0.0079) | -0.0053  (0.0077) |
| White-collar low | -0.0004  (0.0066) | -0.0005  (0.0062) |
| White-collar high | -0.0161**  (0.0047) | -0.0145*  (0.0073) |
| **Country control variables** |  |  |
| Unemployment rate | 0.0013  (0.0039) | 0.0032  (0.0036) |
| Female employment | 0.0084*  (0.0036) | 0.0101*  (0.0034) |
| Women gender role attitudes | 0.0015  (0.0014) | 0.0015  (0.0014) |
| Men gender role attitudes | 0.0009  (0.0013) | 0.0047*  (0.0023) |
| N couple-months | 1.168.164 | 538.061 |
| N couples | 36.596 | 16.931 |

Note: * 0.05 ** 0.01 *** 0.001; Blue-collar low skilled(ISCO 8-9), Blue-collar high skilled (ISCO 6-7), White-collar low skilled (ISCO 4-5), White-collar high skilled (ISCO 1-3)

**S1.2 Labour supply increase of women by presence and age of children (unemployment spells 3 to 6 months)**

| **Explanatory variables** | **Sample A** | | **Sample B** | |
| --- | --- | --- | --- | --- |
| Unemployment | 0.0412***  (0.0123) | 0.0451***  (0.0112) | 0.0380  (0.0292) | 0.0398  (0.0206) |
| Mother | 0.0471***  (0.0086) |  | -0.0042  (0.0078) |  |
| Unemployment*Mother | -0.0152  (0.0183) |  | -0.0025  (0.0304) |  |
| Child 0 to 3 |  | -0.0631***  (0.0187) |  | -0.0343***  (0.0143) |
| Child 4 to 6 |  | 0.0301*  (0.0120) |  | -0.0096  (0.0079) |
| Child 7 to 12 |  | 0.0151  (0.0077) |  | -0.0112***  (0.0050) |
| Unemployment*0 to 3 |  | -0.0049  (0.0363) |  | 0.0178  (0.0411) |
| Unemployment*4 to 6 |  | -0.0232  (0.0184) |  | 0.0385  (0.0579) |
| Unemployment*7 to 12 |  | -0.0272  (0.0235) |  | -0.0403  (0.0342) |
| N couple-months | 1.168.164 | | 538.061 | |
| N couples | 36.596 | | 16.931 | |

Note: * 0.05 ** 0.01 *** 0.001; the models include the control variables at individual, household and country level.

**S1.3 Labour supply increase of women by (general) childcare availability (unemployment spells 3 to 6 months)**


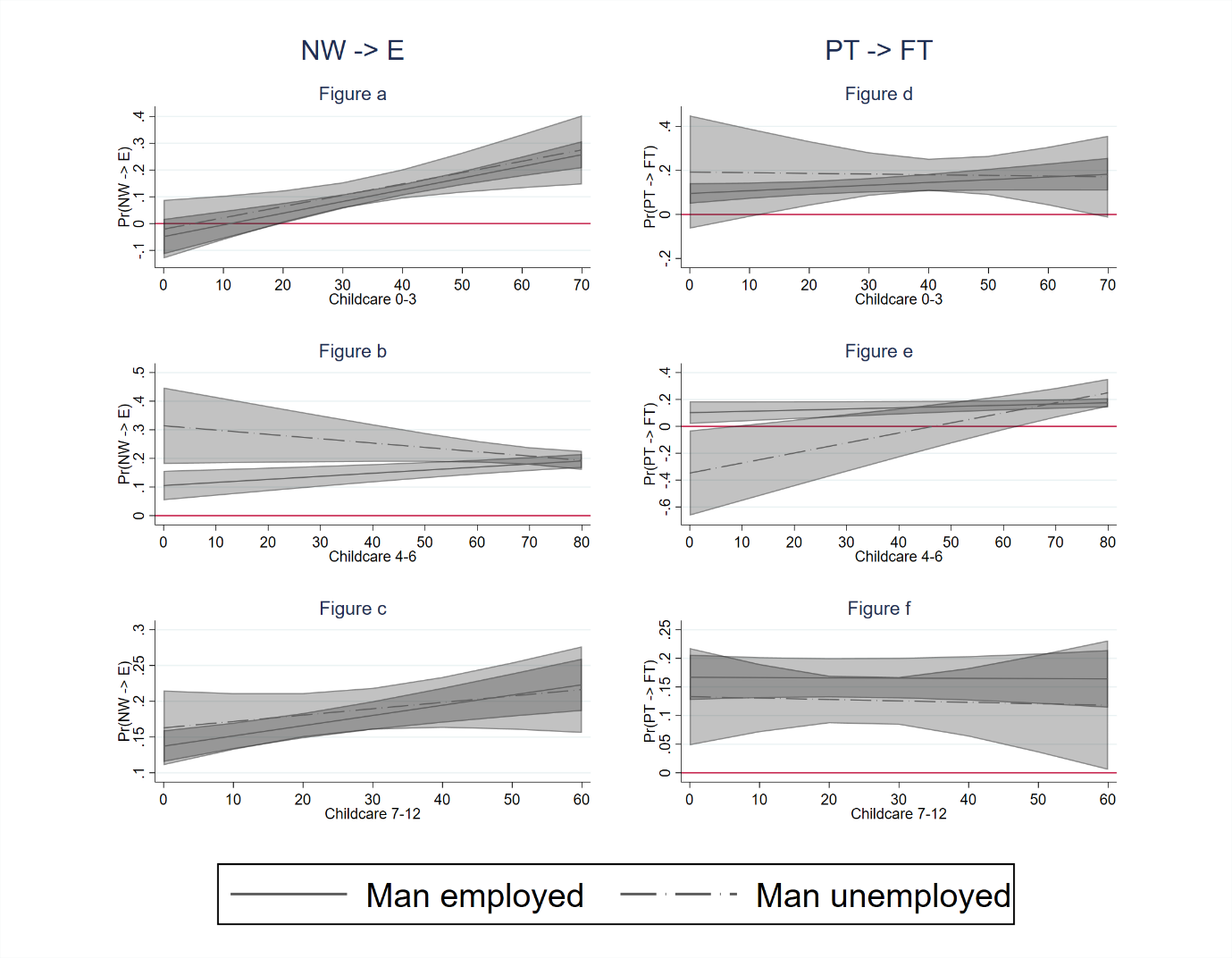


Note: 83% confidence intervals; the models include the control variables at individual, household and country level

**S1.4 Labour supply increase of women by (part-time) childcare availability (unemployment spells 3 to 6 months)**


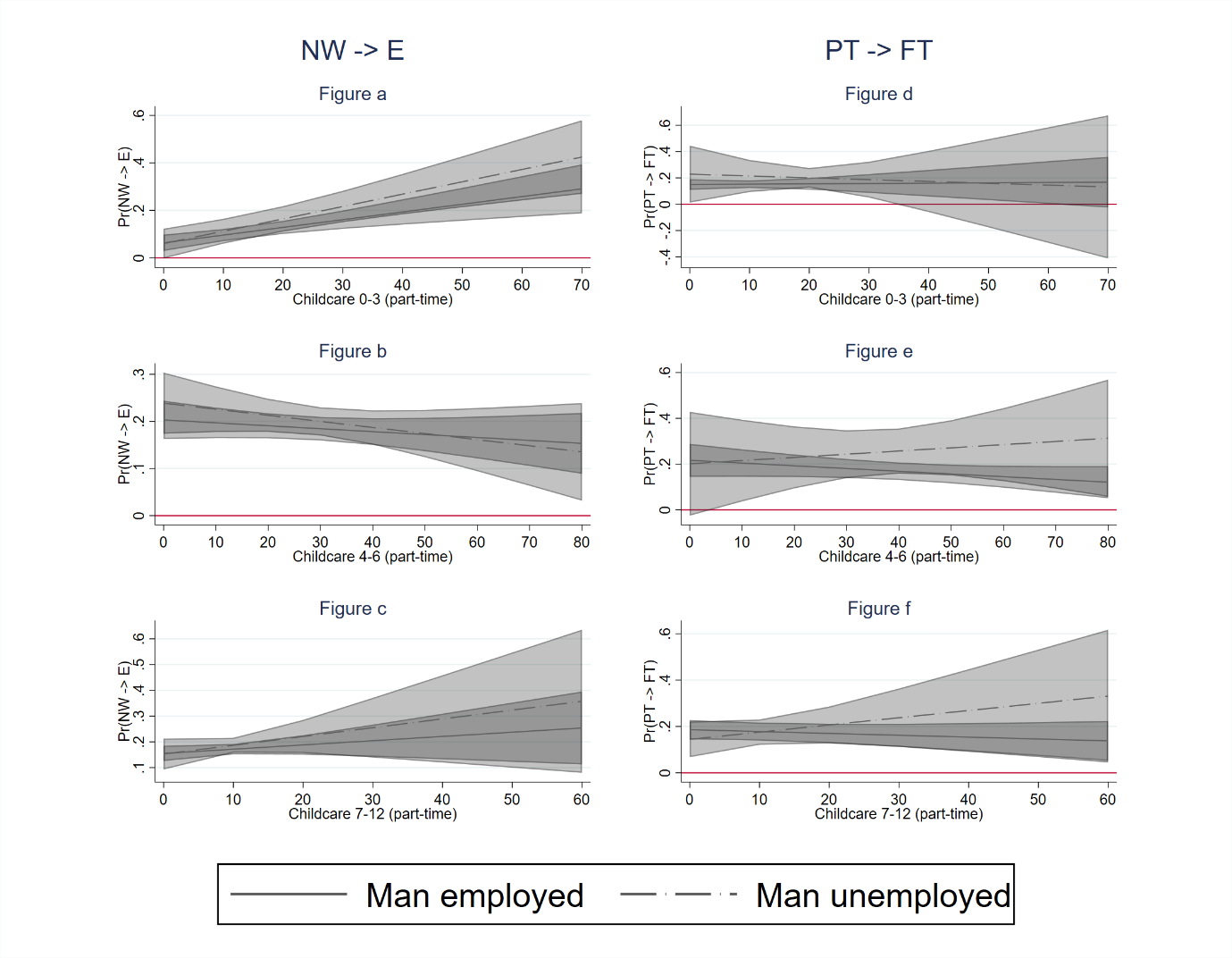


Note: 83% confidence intervals; the models include the control variables at individual, household and country level

**S1.5 Labour supply increase of women by (full-time) childcare availability (unemployment spells 3 to 6 months)**


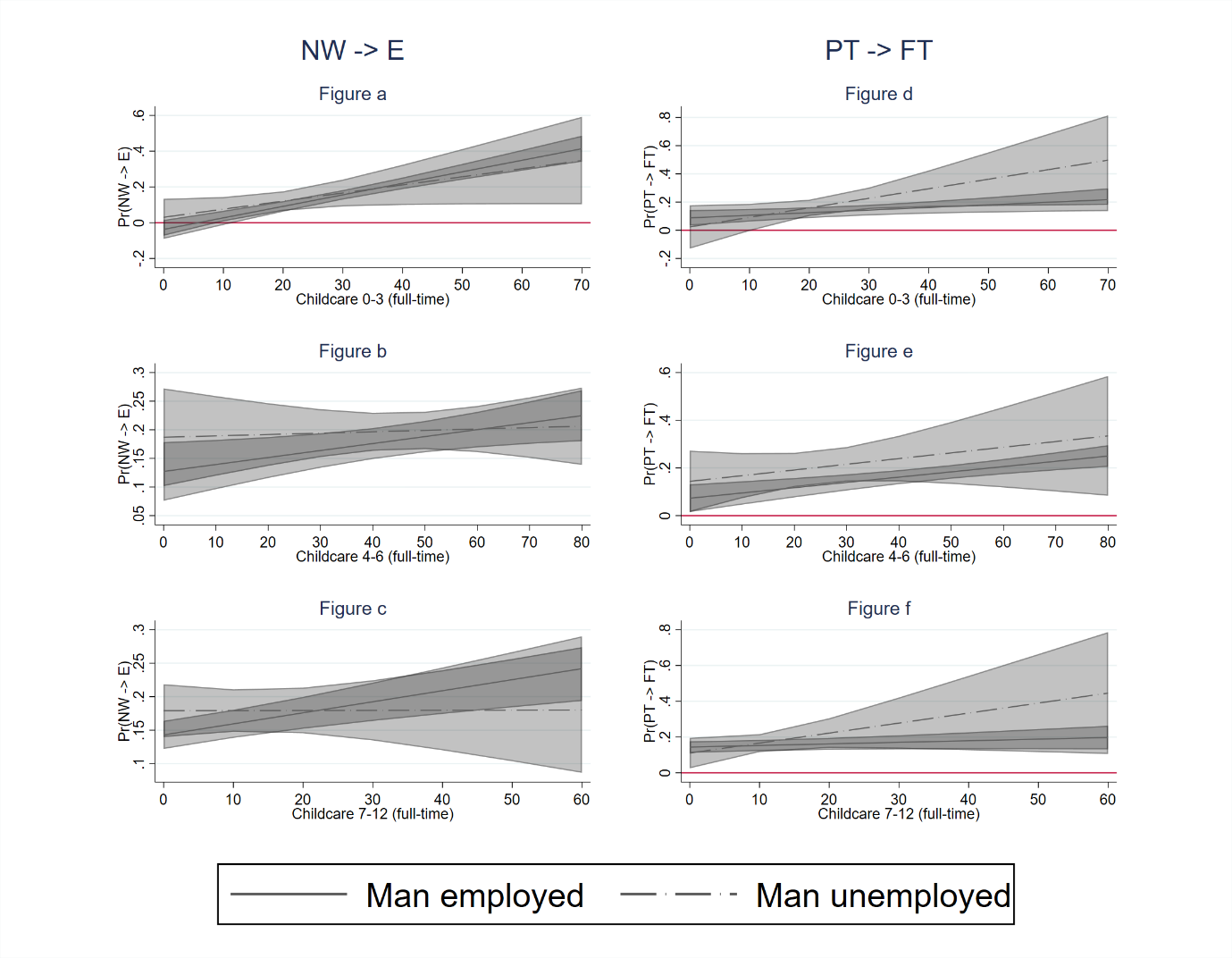


Note: 83% confidence intervals; the models include the control variables at individual, household and country level

**S1.6 Labour supply increase of women by NRRs (unemployment spells 3 to 6 months)**

| **Explanatory variables** | **Sample A** | **Sample B** |
| --- | --- | --- |
| **NRRs: 33%(part-time) and 67% (full-time)** | | |
| Unemployment | 0.0368  (0.0209) | 0.0668  (0.0500) |
| NRRs | 0.0001  (0.0004) | 0.0008  (0.0011) |
| **NRRs: 25%(part-time) and 50% (full-time)** | | |
| Unemployment | 0.0389  (0.0257) | 0.0588  (0.0561) |
| NRRs | 0.0001  (0.0004) | 0.0005  (0.0011) |
| **NRRs: 50%(part-time) and 100% (full-time)** | | |
| Unemployment | 0.0360***  (0.0167) | 0.0501  (0.0392) |
| NRRs | 0.0003  (0.0000) | 0.0005  (0.0010) |
| **NRRs: 75%(part-time) and 150% (full-time)** | | |
| Unemployment | 0.0364  (0.0323) | -0.0275  (0.0433) |
| NRRs | 0.0001  (0.0004) | -0.0014  (0.0010) |
| **NRRs: 100%(part-time) and 200% (full-time)** | | |
| Unemployment | 0.0404  (0.0393) | -0.0404  (0.0459) |
| NRRs | 0.0001  (0.0005) | -0.0015  (0.0010) |
| N couple-months | 1.168.164 | 538.061 |
| N couples | 36.596 | 16.931 |

Note: * 0.05 ** 0.01 *** 0.001; the models include the control variables at individual, household and country level.

**S1.7 Labour supply increase of women by MTRs (unemployment spells 3 to 6 months)**

| **Explanatory variables** | **Sample A** |  | **Sample B** |
| --- | --- | --- | --- |
|  | **NW - 33%** | **NW- 67%** | **33% -67%** |
| Unemployment | 0.0575*  (0.0238) | 0.1006**  (0.0391) | 0.0371  (0.0391) |
| MTRs | 0.0020***  (0.0003) | 0.0036***  (0.0006) | 0.0006  (0.0004) |
| Unemployment*MTRs | -0.0011  (0.0006) | -0.0027**  (0.0011) | -0.0002  (0.0010) |
|  | **NW - 25%** | **NW - 50%** | **25% -50%** |
| Unemployment | 0.0410  (0.0222) | 0.0753**  (0.0285) | 0.0413  (0.0241) |
| MTRs | 0.0014***  (0.0002) | 0.0026***  (0.0004) | 0.0005  (0.0003) |
| Unemployment*MTRs | -0.0005  (0.0004) | -0.0016**  (0.0007) | -0.0003  (0.0005) |
|  | **NW - 50%** | **NW - 100%** | **50 - 100%** |
| Unemployment | 0.0917**  (0.0284) | 0.1326*  (0.0548) | 0.0440  (0.0558) |
| MTRs | 0.0029***  (0.0005) | 0.0054***  (0.0008) | -0.0005  (0.0015) |
| Unemployment*MTRs | -0.0022**  (0.0008) | -0.0036**  (0.0014) | -0.0002  (0.0014) |
|  | **NW - 75%** | **MNW - 150%** | **75 - 150%** |
| Unemployment | 0.0929**  (0.0287) | 0.1360*  (0.0554) | 0.0141  (0.0505) |
| MTRs | 0.0029***  (0.0005) | 0.0055***  (0.0007) | -0.0002  (0.0015) |
| Unemployment*MTRs | -0.0023**  (0.0008) | -0.0037**  (0.0015) | 0.0006  (0.0012) |
|  | **NW - 100%** | **NW- 200%** | **100 - 200%** |
| Unemployment | 0.0826  (0.0433) | 0.0973  (0.0676) | 0.0800  0.0935 |
| MTRs | 0.0044***  (0.0004) | 0.0078***  (0.0009) | 0.0039*  (0.0018) |
| Unemployment*MTRs | -0.0021  (0.0011) | -0.0025  (0.0018) | -0.0010  (0.0022) |
| N couple-months | 1.168.164 | | 538.061 |
| N couples | 36.596 | | 16.931 |

Note: * 0.05 ** 0.01 *** 0.001; the models include the control variables at individual, household and country level.
