# Supplementary material for: His unemployment, her response, and the moderating role of welfare policies in European countries. Results from a preregistered study
Source: PLoS One. 2024 Aug 20;19(8):e0306964. doi: 10.1371/journal.pone.0306964 (PMC11335131; doi:10.1371/journal.pone.0306964)
Supplement: S2 Appendix — (DOCX) [file pone.0306964.s007.docx]

**S2. Appendix. Results for models restricted to unemployment spells lasting longer than 6 months**

**S2.1. Labour supply increase of women (unemployment spells longer than 6 months)**

| **Explanatory variables** | **Sample A** | **Sample B** |
| --- | --- | --- |
| Unemployment | 0.0717***  (0.0132) | 0.0930***  (0.0187) |
| **Household characteristics** |  |  |
| Married *(ref. cohabiting)* | -0.0357***  (0.0101) | -0.0095*  (0.0042) |
| Number of children | -0.0129***  (0.0035) | -0.0001  (0.0028) |
| Child aged 0 to 3 | -0.0636**  (0.0184) | -0.0342*  (0.0142) |
| Child aged 4 to 6 | 0.0304*  (0.0120) | -0.0098  (0.0077) |
| Child aged 7 to 12 | 0.0149*  (0.0076) | -0.0122**  (0.0047) |
| Income (*ref. Quintile 1*) |  |  |
| Quintile 2 | 0.0221***  (0.0060) | -0.0068  (0.0085) |
| Quintile 3 | 0.0559***  (0.0089) | -0.0002  (0.0095) |
| Quintile 4 | 0.0824***  (0.0109) | 0.0174  (0.0111) |
| Quintile 5 | 0.0890***  (0.0109) | 0.0277*  (0.0110) |
| **Female characteristics** |  |  |
| Age | -0.0030***  (0.0005) | -0.0028***  (0.0005) |
| Education *(ref. low)* |  |  |
| Medium | 0.0368***  (0.0082) | 0.0081  (0.0072) |
| High | 0.106***  (0.0109) | 0.0288**  (0.0093) |
| Occupation (*ref. Blue low*) | - |  |
| Blue-collar high |  | 0.0226*  (0.0109) |
| White-collar low |  | 0.0221*  (0.0107) |
| White-collar high |  | 0.0507***  (0.0141) |
| **Male characteristics** |  |  |
| Age | -0.0025***  (0.0004) | -0.0008  (0.0006) |
| Education (*ref. low*) |  |  |
| Medium | 0.0062  (0.0058) | -0.0074  (0.0081) |
| High | -0.0078  (0.0051) | -0.01119  (0.0062) |
| Occupation (*ref. Blue low*) |  |  |
| Blue-collar high | -0.0062  (0.0079) | -0.0062  (0.0076) |
| White-collar low | 0.0002  (0.0068) | -0.0014  (0.0063) |
| White-collar high | -0.0173**  (0.0052) | -0.0145*  (0.0072) |
| **Country control variables** |  |  |
| Unemployment rate | 0.0016  (0.0038) | 0.0037  (0.0037) |
| Female employment | 0.0085*  (0.0036) | 0.0101*  (0.0034) |
| Women gender role attitudes | 0.0015  (0.0014) | 0.0016  (0.0015) |
| Men gender role attitudes | 0.0010  (0.0013) | 0.0047*  (0.0022) |
| N couple-months | 1.186.926 | 542.707 |
| N couples | 36.601 | 16.931 |

Note: * 0.05 ** 0.01 *** 0.001; Blue-collar low skilled(ISCO 8-9), Blue-collar high skilled (ISCO 6-7), White-collar low skilled (ISCO 4-5), White-collar high skilled (ISCO 1-3)

**S2.2.  Labour supply increase of women by presence and age of children (unemployment spells longer than 6 months)**

| **Explanatory variables** | **Sample A** | | **Sample B** | |
| --- | --- | --- | --- | --- |
| Unemployment | 0.0617***  (0.0180) | 0.0728***  (0.0157) | 0.0503*  (0.0230) | 0.0709***  (0.0191) |
| Mother | 0.0509***  (0.0095) |  | -0.0059  (0.0075) |  |
| Unemployment*Mother | 0.0192  (0.0175) |  | 0.0748***  (0.0208) |  |
| Child 0 to 3 |  | -0.0632***  (0.0186) |  | -0.0347***  (0.0141) |
| Child 4 to 6 |  | 0.0303*  (0.0121) |  | -0.0100  (0.0080) |
| Child 7 to 12 |  | 0.0148  (0.0078) |  | -0.0126***  (0.0048) |
| Unemployment*0 to 3 |  | -0.0238  (0.0247) |  | 0.0649  (0.1045) |
| Unemployment*4 to 6 |  | 0.0023  (0.0216) |  | 0.0217  (0.0544) |
| Unemployment*7 to 12 |  | 0.0074  (0.0219) |  | 0.0402  (0.0324) |
| N couple-months | 1.186.926 | | 542.707 | |
| N couples | 36.601 | | 16.931 | |

Note: * 0.05 ** 0.01 *** 0.001; the models include the control variables at individual, household and country level

**S2.3 Labour supply increase of women by (general) childcare availability (unemployment spells longer than 6 months)**

**
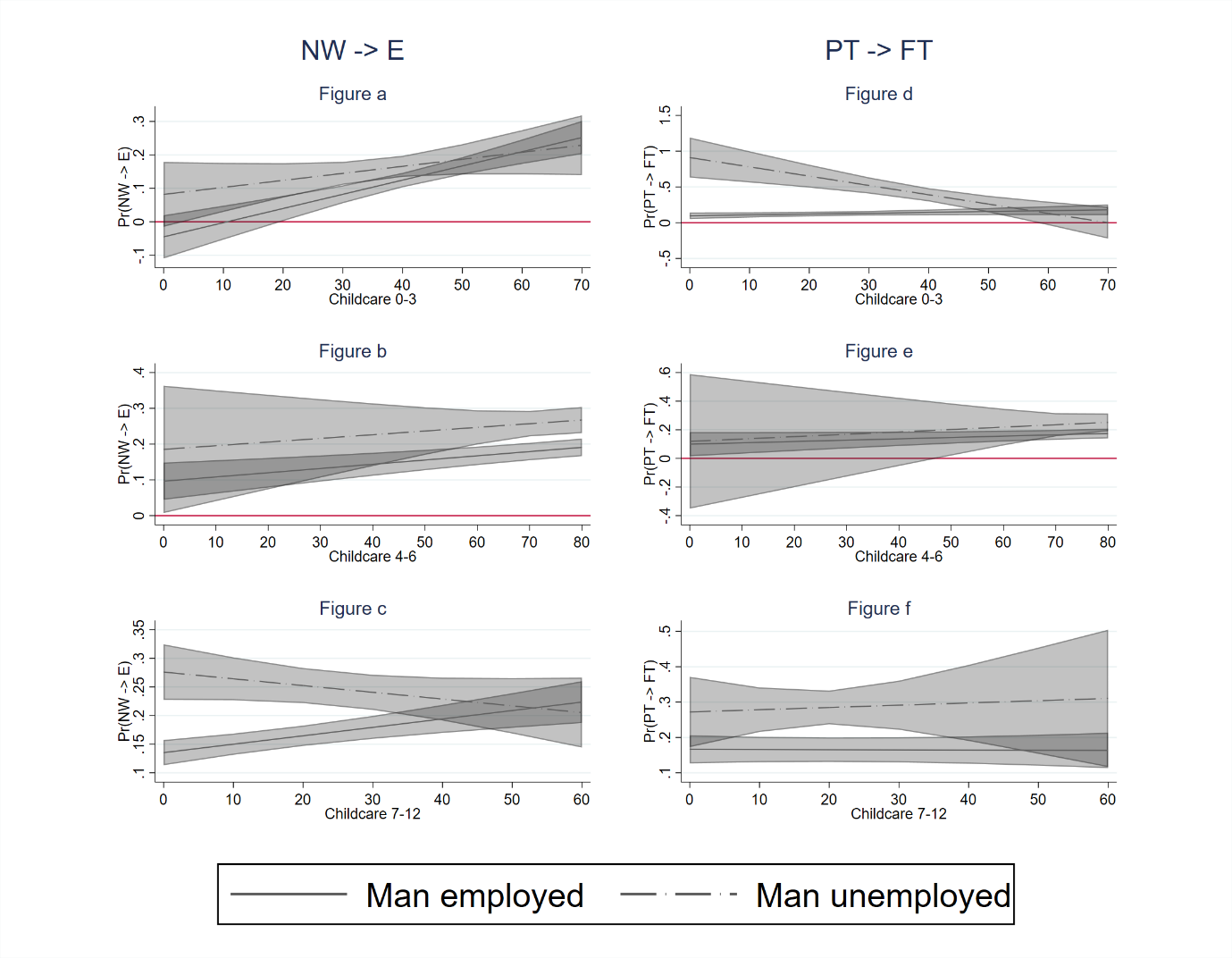
**

Note: 83% confidence intervals; the models include the control variables at individual, household and country level

**S2.4 Labour supply increase of women by (part-time) childcare availability (unemployment spells longer than 6 months)**

**
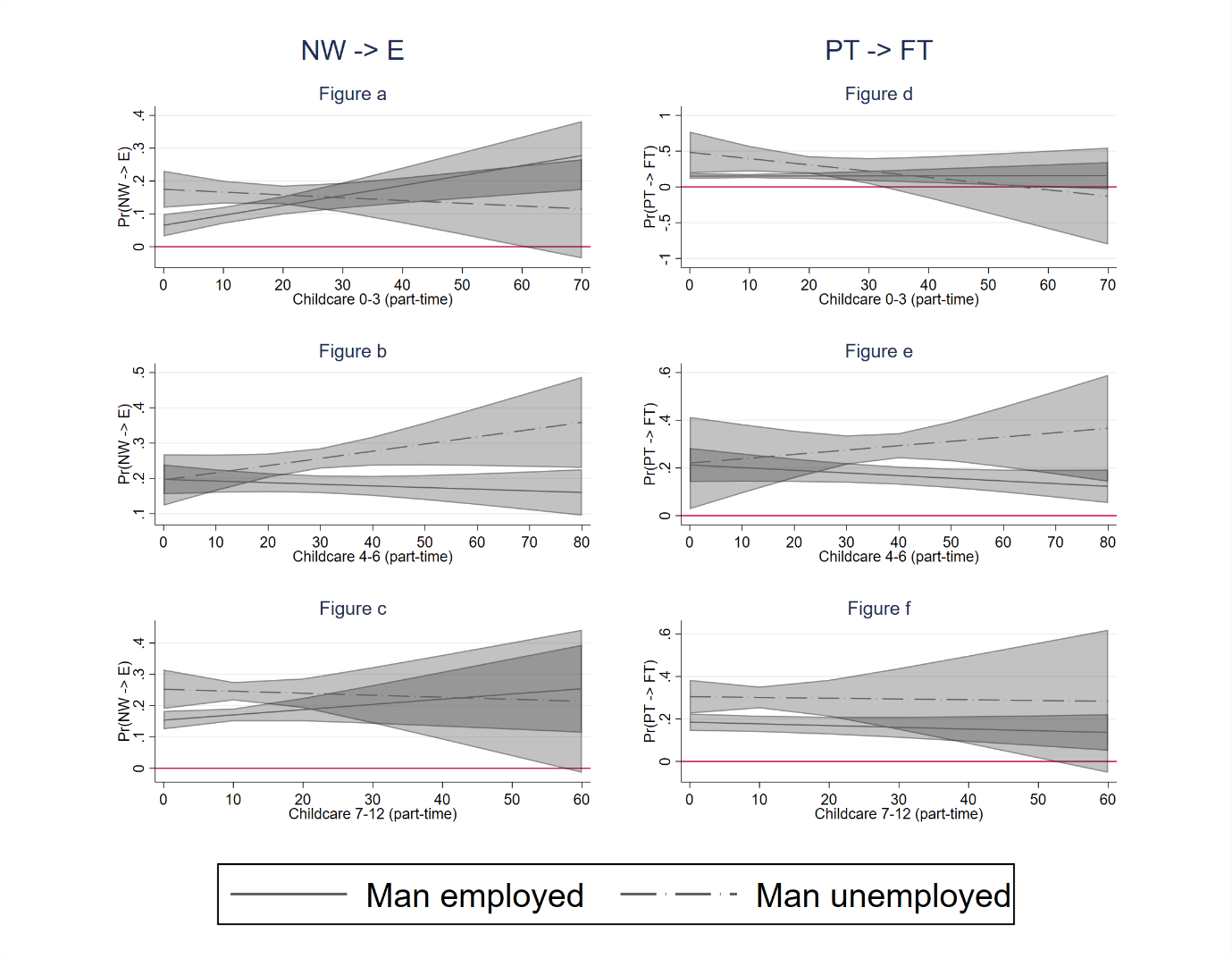
**

Note: 83% confidence intervals; the models include the control variables at individual, households and country level

**S2.5 Labour supply increase of women by (full-time) childcare availability (unemployment spells longer than 6 months)**


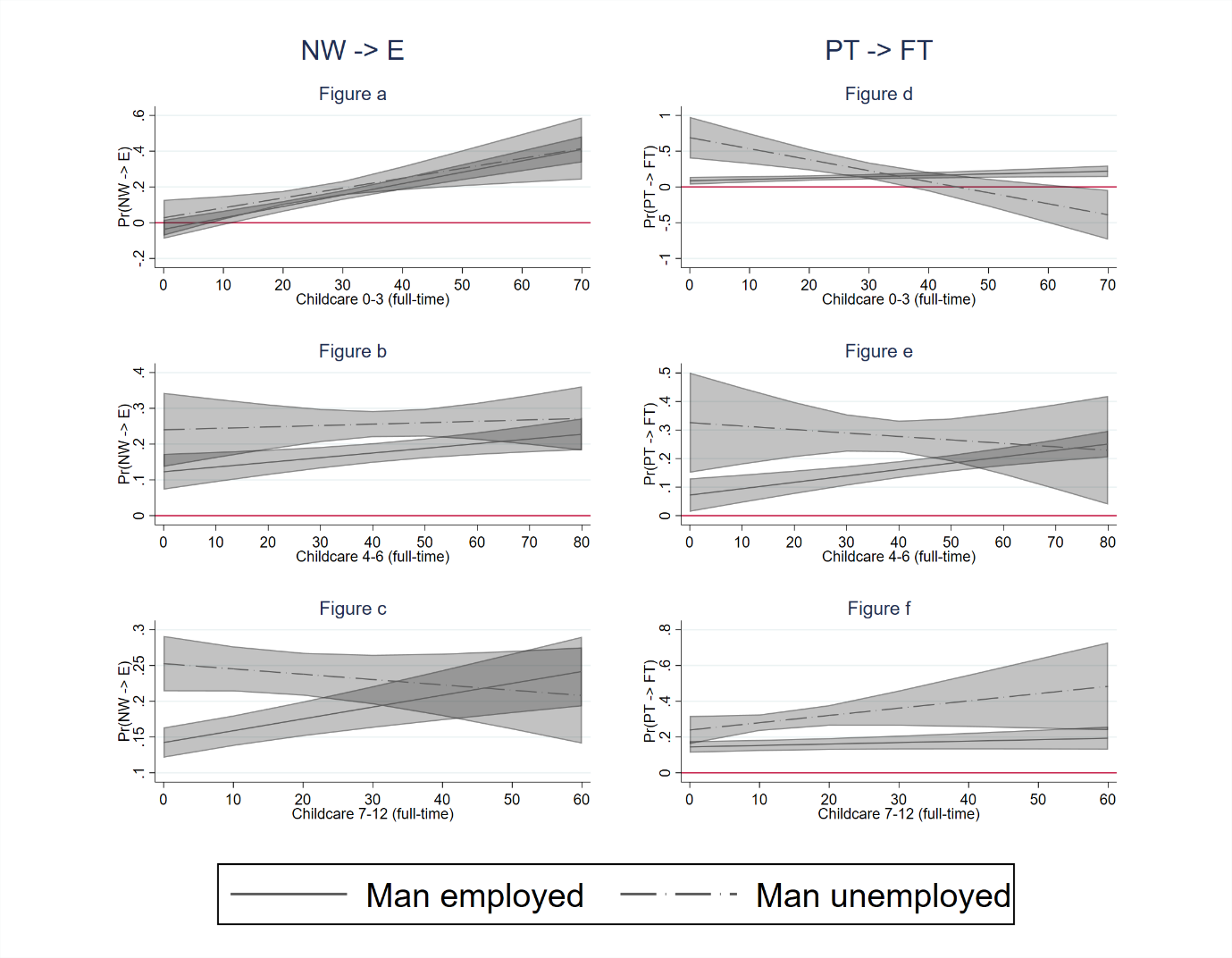


Note: 83% confidence intervals; the models include the control variables at individual, households and country level

**Table 2.6 Labour supply increase of women by NRRs (unemployment spells longer than 6 months)**

| **Explanatory variables** | **Sample A** | **Sample B** |
| --- | --- | --- |
| **NRRs: 33%(part-time) and 67% (full-time)** | | |
| Unemployment | 0.0882**  (0.0294) | 0.1532**  (0.0485) |
| NRRs | 0.0004  (0.0005) | 0.0017  (0.0012) |
| **NRRs: 50%(part-time) and 100% (full-time)** | | |
| Unemployment | 0.0881**  (0.0359) | 0.1326**  (0.0589) |
| NRRs | 0.0003  (0.0005) | 0.0009  (0.0012) |
| **NRRs: 25%(part-time) and 50% (full-time)** | | |
| Unemployment | 0.0829**  (0.0242) | 0.1461**  (0.0346) |
| NRRs | 0.0003  (0.0004) | 0.0019  (0.0010) |
| **NRRs: 75%(part-time) and 100% (full-time)** | | |
| Unemployment | 0.1028*  (0.0447) | 0.1168  (0.0616) |
| NRRs | 0.0005  (0.0006) | 0.0005  (0.0011) |
| **NRRs: 100%(part-time) and 200% (full-time)** | | |
| Unemployment | 0.1223*  (0.0517) | 0.1215  (0.0623) |
| NRRs | 0.0007  (0.0006) | 0.0006  (0.0011) |
| N couple-months | 1.186.926 | 542.707 |
| N couples | 36.601 | 16.931 |

Note: * 0.05 ** 0.01 *** 0.001; the models include the control variables at individual, household and country level

**Table 2.7 Labour supply increase of women by MTRs (unemployment spells longer than 6 months)**

| **Explanatory variables** | **Sample A** | | **Sample B** |
| --- | --- | --- | --- |
|  | **NW - 33%** | **NW - 67%** | **33% -67%** |
| Unemployment | 0.0919***  (0.0250) | 0.1007***  (0.0390) | 0.0090  (0.0344) |
| MTRs | 0.0019***  (0.0003) | 0.0036***  (0.0006) | 0.0006  (0.0004) |
| Unemployment*MTRs | -0.0009*  (0.0004) | -0.0027*  (0.00010) | 0.0020**  (0.0008) |
|  | **NW - 25%** | **NW - 50%** | **25% -50%** |
| Unemployment | 0.0985***  (0.0359) | 0.0753***  (0.0284) | 0.0532**  (0.0178) |
| MTRs | 0.0014***  (0.0002) | 0.0025***  (0.0007) | 0.0005  (0.0003) |
| Unemployment*MTRs | -0.0008  (0.0004) | -0.0016*  (0.00007) | 0.0009*  (0.0004) |
|  | **NW - 50%** | **NW - 100%** | **50 - 100%** |
| Unemployment | 0.1003***  (0.0195) | 0.1313***  (0.0547) | 0.0443  (0.0495) |
| MTRs | 0.0029***  (0.0004) | 0.0520***  (0.0007) | -0.0003  (0.0014) |
| Unemployment*MTRs | -0.0016*  (0.0005) | -0.0034*  (0.00014) | 0.0013  (0.0013) |
|  | **NW - 75%** | **NW - 150%** | **75 - 150%** |
| Unemployment | 0.1045***  (0.0198) | 0.1346***  (0.0554) | 0.0601  (0.0472) |
| MTRs | 0.0030***  (0.0005) | 0.0053***  (0.0007) | -0.0001  (0.0014) |
| Unemployment*MTRs | -0.0017*  (0.0005) | -0.0035*  (0.00014) | 0.0009  (0.0012) |
|  | **NW - 100%** | **NW - 200%** | **100 - 200%** |
| Unemployment | 0.1054***  (0.0226) | 0.1261***  (0.0525) | 0.1355  (0.0929) |
| MTRs | 0.0043***  (0.0004) | 0.0078***  (0.0008) | 0.040*  (0.0017) |
| Unemployment*MTRs | -0.0016*  (0.0006) | -0.0023  (0.00017) | -0.0010  (0.0024) |
| N couple-months | 1.186.926 | | 542.707 |
| N couples | 36.601 | | 16.931 |

Note: * 0.05 ** 0.01 *** 0.001; the models include the control variables at individual, household and country level
