# Supplementary material for: His unemployment, her response, and the moderating role of welfare policies in European countries. Results from a preregistered study
Source: PLoS One. 2024 Aug 20;19(8):e0306964. doi: 10.1371/journal.pone.0306964 (PMC11335131; doi:10.1371/journal.pone.0306964)
Supplement: Attachment — Submitted filename: Answers to reviewers comments.docx [file pone.0306964.s008.docx]

**We would like to thank both reviewers for their comments and suggestions. Below we have provided a point-by-point answer to each comment and suggestion received.**

**Reviewer #1:** This study explores the dynamic response of women's labor supply to their male partners' unemployment across 24 EU member states and the UK during the period 2009-2019. Using longitudinal data from EU-SILC and policy simulation models EUROMOD and UKMOD, the study investigates how welfare policies, particularly childcare and tax-benefit systems, influence women's decision to enter the labor market or increase working hours in response to economic shocks affecting their households.

The alleged innovation of this study lies in its comprehensive approach to examining the moderating effects of welfare policies on the labor supply decisions of women facing partners' unemployment. The research stands out for its application of harmonized data across multiple European countries and its methodological approach in using tax-benefit simulation models to analyze both the extensive (entering employment) and intensive (increasing working hours) margins of labor supply. This perspective provides a nuanced understanding of women's labor market behavior in varying policy environments.

The authors deserve commendation for their effort in harmonizing complex longitudinal data across diverse national contexts, enabling a comparative analysis of labor supply responses under different welfare regimes. The scope of the research is particularly commendable, as it addresses both the extensive and intensive margins of labor market participation, enriching the discourse on gender, labor, and social policy.

Answer: Thank you!

While the study is methodologically robust, certain necessary assumptions warrant attention:

The use of predefined household structures may not capture the full diversity of family types, potentially limiting the generalizability of findings to less conventional or evolving family models.

Answer: We fully agree that family types are highly diverse and that this study looks only at one type of families. Still such families are quite numerous in the EU (which is the region we cover), especially when children are small, and the mechanism we investigate (the added worker effect) has been widely addressed, especially in economic literature. With this study we contribute exactly to this strand of the literature by incorporating the role of the specific policies for her response to his unemployment. This is how the study was designed and preregistered after it was evaluated and accepted by several external reviewers in Plos One. However, we agree that it does not capture full diversity of family types and that the added worker affect could also be studied for same-sex couples or female breadwinner couples. Unfortunately our data would not allow us to examine it as the number of such couples in our data is too small. We admit this limitation in the paper (please see lines 70-72 and 799-803). We believe, however, our study has a potential to stimulate research for these couples on larger datasets with the use of the methodology we proposed.

Fixed income levels and standardized employment scenarios (full-time, part-time, unemployed) may oversimplify the real-life complexities and variability of employment conditions and earnings.

Answer: Unfortunately the EU-SILC data does not allow for accounting for more nuanced employment statuses. As every dataset it has its advantages and disadvantages. The advantage is that it provides monthly retrospective data on employment for all EU member states. Its disadvantage is that the monthly calendar data provides only information on the very general employment status of the individuals (i.e. full-time employed; part-time employed, unemployed, inactive) of respondents. Consequently, using this dataset we are only able to study his unemployment and her transitions into activity and full-time employment. Having said that, we would like to mention that we have also conducted another study, which complements this one, and in which we were able to cover more diverse employment transitions (e.g. the changes in the number of her working hours in reaction to his entry into unemployment or nonstandard employment) (see Matysiak et al 2024). The study was conducted based on EU LFS data, which provided more nuanced data on employment but had other limitations (e.g. much shorter observation span than EU-SILC). All in all, we are convinced that the two studies provide a comprehensive overview of the added-worker effect in the EU and the role of the policies for moderating her response to the worsening in his employment situation which can stimulate further research on the topic.

Assuming 100% benefit take-up ignores the real-world challenges and barriers households may face in accessing entitled benefits, possibly overstating the protective capacity of welfare systems.

Answer: We agree that assuming a 100% benefit take-up and setting static percentages of average wages constitute limitations of the study. Both limitations stem from the design of the microsimulation tool EUROMOD we employ to estimate the net replacement rates (NRR) and marginal tax rates (MRTs). As mentioned in the manuscript, by design EUROMOD assumes a 100% benefit take-up. We acknowledge this limitation in the manuscript and have stressed its implications (please see line 814-820).

Setting static percentages of average wages for income estimations may not accurately reflect the diverse economic realities faced by individuals across different sectors and regions.

Answer: Estimating the MTRs and NRRs based on the actual income reported by the couples in our dataset is not possible. This is because the EU-SILC data as made available for research purposes is not compatible with EUROMOD. Consequently, we relied on the hypothetical household tool (HHT) provided by EUROMOD to generate hypothetical household data, which was later on used to estimate the MTRs and NRRs. In generating the hypothetical data the HHT allows users to specify wage variables as percentages of predefined country-specific average wages. These predefined wage levels cannot be adjusted so as to account for differences between regions or sectors. Moreover, the number of households generated grows exponentially with the number of categories assumed by each variable. Thus, we aimed at reducing the number of the categories to a minimum. As a result, we defined the households using static percentages of country-specific average wages. However, in order to test the sensitivity of the results we have varied the wage-thresholds used. Additionally, as already mentioned, we have acknowledged this limitation in the manuscript (please see lines 814-820). Finally, we would like to stress that this problem was raised by a reviewer of our preregistered study protocol which was accepted after our explanations.

While the study uses updated policy information, there may be delays in reflecting recent policy changes or economic shifts, impacting the timeliness and relevance of the analysis.

Answer: We agree that there may be delays in the effects of recent policy changes and we are not able to grasp these effects with our study. However, this problem is not specific to our analysis only, but rather a common problem across all studies using large-scale survey data. Data available for the analysis always comes with a delay and empirical analyses are never able to grasp all the effects of recent policies. Furthermore, the period 2020-2022 was very specific due to the pandemic. Therefore, we decided to use the most recent data, but leave out the pandemic period. Finally, we did it exactly as planned in the (reviewed and accepted) pre-registration, which we were obliged to follow.

In conclusion, while the study provides significant contributions to understanding the interplay between unemployment, gender dynamics, and welfare policy, I would recommend that the authors better emphasise the limitations of their assumptions.

Answer: Thank you, we stressed the limitations of our assumptions in the paper as pointed out in the answers above.

**Reviewer #3:** This is a well-done research paper, clearly addressing the question it sets out to answer: Do women increase their labour supply in response to partner's unemployment and which factors mitigate this response?

The paper strikes me as well-developed and clearly written, using good data and clearly communicating an adequate empirical strategy and can be published at this point.

The strongest aspect of this work is the careful combination of multiple data sources that were combined to clearly address each of the hypotheses.

Answer: Thank you!

What I consider to be the two biggest remaining weaknesses are:

1) potential anticipation of the partners unemployment, which would affect the validity of the empirical estimates: however the authors are aware of this shortcoming and honest about it in the conclusion. As this is hard to correct, I think the honest acknowledgement of this problem is the best the authors can do.

Answer: Yes, we acknowledge this problem in the conclusions.

2) the unidirectional measurement of the labour market response: what I mean by this is that I suspect that there is a third unmeasured channel, and that is that if both partners are working and one of them loses employment, the female partner might become less likely to reduce her labour supply. This channel is ruled out by restricting the sample to previously unemployed or part-time working women.

Answer: Thank you for this suggestion. Incorporating it at that stage would be against the rules of the preregistration and would substantially expand the paper. We will consider it however in our future research.

As said, I consider the paper publishable at this point and comment 2) only serves as a potential avenue if the authors end up reworking the paper, as it does adequately answer the questions it set out to answer at this point.

Some minor remarks:

- a short normative discussion on advantages and disadvantages of having women increase their labour supply in response to partners income shocks would help make sense of the results a bit more (it seems like there are conflicting objectives between wanting to increasing female labour supply and making sure families are adequately insured and do not have to forego their childcare priorities etc. here). Also

Answer: We agree that there is a conflict between the advantages and disadvantages of an increase in women's labour supply. We have highlighted these in the section on conclusions and discussions of the manuscript (please see lines 785-797)

- the one result where i would have a slightly different reading from the authors is with respect to childcare: they seem to read their finding that there is only an effect for mothers with children age 0-3 as showing that childcare does not have much of an effect at all, but my expectation would have been that there would probably mainly be an effect at those ages, so I am inclined to read the childcare results as being more meaningful than the authors

Answer: In light of your observation we have highlighted the effect we identified for mothers with young children (please see lines 737-742).

- a brief table with a very simple overview of the different channels (partners unemployment, interacted with marginal tax rate, interaced with childcare etc.) and the direction and strength of their effects on non-working and part-time working mothers to summarize all the results in one simple and easily place would serve the paper in terms of science communication and in terms of giving policymakers an easily readable tool of what matters for increasing female labour supply.

Answer: We have included such a table in the conclusions and discussion section of the manuscript.

I recognize that the authors cannot share their data as normally requested by PLOS ONE. Providing a github with the code with which the analysis was done would be good in this case.

Answer: The code, as well as the country-level data used in the analyses is publicly available here: <https://github.com/LabFam/rEUsilience> .

References:

Matysiak, Anna, Anna Kurowska, and Alina Maria Pavelea. 2024. *Adjustments in women’s labour force participation in response to the*. rEUsilience Working Paper Series No 6.
