# Supplementary material for: His unemployment, her response, and the moderating role of welfare policies in European countries. Results from a preregistered study
Source: PLoS One. 2024 Aug 20;19(8):e0306964. doi: 10.1371/journal.pone.0306964 (PMC11335131; doi:10.1371/journal.pone.0306964)
Supplement: Peer review history

# Peer review: Decision Letter 0

**DOI:** 10.1371/journal.pone.0306964.r001

**Authors:** Jordi Gumà

2 May 2024

PONE-D-24-04533His unemployment, her response, and the moderating role of welfare policies in European countriesPLOS ONE

Dear Dr. Matysiak,

Thank you for submitting your manuscript to PLOS ONE. After careful consideration, we feel that it has merit but does not fully meet PLOS ONE’s publication criteria as it currently stands. Therefore, we invite you to submit a revised version of the manuscript that addresses the points raised during the review process.

As I mentioned in a previous email, we were awaiting the comments of a third reviewer. You will see that the third reviewer's comments are more in line with the first reviewer's comments and my own assessment of your work. Please consider responding to the few issues proposed by these reviewers. 

Please submit your revised manuscript by Jun 14 2024 11:59PM. If you will need more time than this to complete your revisions, please reply to this message or contact the journal office at plosone@plos.org. When you're ready to submit your revision, log on to https://www.editorialmanager.com/pone/ and select the 'Submissions Needing Revision' folder to locate your manuscript file.

Please include the following items when submitting your revised manuscript:

- A rebuttal letter that responds to each point raised by the academic editor and reviewer(s). You should upload this letter as a separate file labeled 'Response to Reviewers'.
- A marked-up copy of your manuscript that highlights changes made to the original version. You should upload this as a separate file labeled 'Revised Manuscript with Track Changes'.
- An unmarked version of your revised paper without tracked changes. You should upload this as a separate file labeled 'Manuscript'.

If you would like to make changes to your financial disclosure, please include your updated statement in your cover letter. Guidelines for resubmitting your figure files are available below the reviewer comments at the end of this letter.

If applicable, we recommend that you deposit your laboratory protocols in protocols.io to enhance the reproducibility of your results. Protocols.io assigns your protocol its own identifier (DOI) so that it can be cited independently in the future. For instructions see: https://journals.plos.org/plosone/s/submission-guidelines#loc-laboratory-protocols. Additionally, PLOS ONE offers an option for publishing peer-reviewed Lab Protocol articles, which describe protocols hosted on protocols.io. Read more information on sharing protocols at https://plos.org/protocols?utm_medium=editorial-email&utm_source=authorletters&utm_campaign=protocols.

We look forward to receiving your revised manuscript.

Kind regards,

Jordi Gumà, Ph.D.

Academic Editor

PLOS ONE

Journal Requirements:

When submitting your revision, we need you to address these additional requirements.

1. Please ensure that your manuscript meets PLOS ONE's style requirements, including those for file naming. The PLOS ONE style templates can be found at 

https://journals.plos.org/plosone/s/file?id=wjVg/PLOSOne_formatting_sample_main_body.pdf and 

https://journals.plos.org/plosone/s/file?id=ba62/PLOSOne_formatting_sample_title_authors_affiliations.pdf

2. Thank you for stating the following financial disclosure: "This project has received funding from the European Union’s Horizon Europe research executive agency (REA) under Grant Agreement No.101060410 as well as the Polish National Agency for Academic Exchange (Polish Returns 2019). "

Please state what role the funders took in the study.  If the funders had no role, please state: ""The funders had no role in study design, data collection and analysis, decision to publish, or preparation of the manuscript."" 

If this statement is not correct you must amend it as needed. 

Please include this amended Role of Funder statement in your cover letter; we will change the online submission form on your behalf.

3. We note that you have indicated that there are restrictions to data sharing for this study. PLOS only allows data to be available upon request if there are legal or ethical restrictions on sharing data publicly. For more information on unacceptable data access restrictions, please see http://journals.plos.org/plosone/s/data-availability#loc-unacceptable-data-access-restrictions. 

Before we proceed with your manuscript, please address the following prompts:

a) If there are ethical or legal restrictions on sharing a de-identified data set, please explain them in detail (e.g., data contain potentially identifying or sensitive patient information, data are owned by a third-party organization, etc.) and who has imposed them (e.g., a Research Ethics Committee or Institutional Review Board, etc.). Please also provide contact information for a data access committee, ethics committee, or other institutional body to which data requests may be sent.

b) If there are no restrictions, please upload the minimal anonymized data set necessary to replicate your study findings to a stable, public repository and provide us with the relevant URLs, DOIs, or accession numbers. For a list of recommended repositories, please see

https://journals.plos.org/plosone/s/recommended-repositories. You also have the option of uploading the data as Supporting Information files, but we would recommend depositing data directly to a data repository if possible.

We will update your Data Availability statement on your behalf to reflect the information you provide.

4. Please include your full ethics statement in the ‘Methods’ section of your manuscript file. In your statement, please include the full name of the IRB or ethics committee who approved or waived your study, as well as whether or not you obtained informed written or verbal consent. If consent was waived for your study, please include this information in your statement as well.

5. Please review your reference list to ensure that it is complete and correct. If you have cited papers that have been retracted, please include the rationale for doing so in the manuscript text, or remove these references and replace them with relevant current references. Any changes to the reference list should be mentioned in the rebuttal letter that accompanies your revised manuscript. If you need to cite a retracted article, indicate the article’s retracted status in the References list and also include a citation and full reference for the retraction notice.

[Note: HTML markup is below. Please do not edit.]

Reviewers' comments:

Reviewer's Responses to Questions

**Comments to the Author**

1. Does the manuscript adhere to the experimental procedures and analyses described in the Registered Report Protocol?

If the manuscript reports any deviations from the planned experimental procedures and analyses, those must be reasonable and adequately justified.

Reviewer #1: Yes

Reviewer #3: Yes

********** 

2. If the manuscript reports exploratory analyses or experimental procedures not outlined in the original Registered Report Protocol, are these reasonable, justified and methodologically sound?

A Registered Report may include valid exploratory analyses not previously outlined in the Registered Report Protocol, as long as they are described as such.

Reviewer #1: Yes

Reviewer #3: Yes

********** 

3. Are the conclusions supported by the data and do they address the research question presented in the Registered Report Protocol?

The manuscript must describe a technically sound piece of scientific research with data that supports the conclusions. The conclusions must be drawn appropriately based on the research question(s) outlined in the Registered Report Protocol and on the data presented.

Reviewer #1: Yes

Reviewer #3: Yes

********** 

4. Have the authors made all data underlying the findings in their manuscript fully available?

The PLOS Data policy requires authors to make all data underlying the findings described in their manuscript fully available without restriction, with rare exception (please refer to the Data Availability Statement in the manuscript PDF file). The data should be provided as part of the manuscript or its supporting information, or deposited to a public repository. For example, in addition to summary statistics, the data points behind means, medians and variance measures should be available. If there are restrictions on publicly sharing data—e.g. participant privacy or use of data from a third party—those must be specified.

Reviewer #1: No

Reviewer #3: Yes

********** 

5. Is the manuscript presented in an intelligible fashion and written in standard English?

*PLOS ONE* does not copyedit accepted manuscripts, so the language in submitted articles must be clear, correct, and unambiguous. Any typographical or grammatical errors should be corrected at revision, so please note any specific errors here.

Reviewer #1: Yes

Reviewer #3: Yes

********** 

6. Review Comments to the Author

Please use the space provided to explain your answers to the questions above. (Please upload your review as an attachment if it exceeds 20,000 characters)

Reviewer #1: This study explores the dynamic response of women's labor supply to their male partners' unemployment across 24 EU member states and the UK during the period 2009-2019. Using longitudinal data from EU-SILC and policy simulation models EUROMOD and UKMOD, the study investigates how welfare policies, particularly childcare and tax-benefit systems, influence women's decision to enter the labor market or increase working hours in response to economic shocks affecting their households.

The alleged innovation of this study lies in its comprehensive approach to examining the moderating effects of welfare policies on the labor supply decisions of women facing partners' unemployment. The research stands out for its application of harmonized data across multiple European countries and its methodological approach in using tax-benefit simulation models to analyze both the extensive (entering employment) and intensive (increasing working hours) margins of labor supply. This perspective provides a nuanced understanding of women's labor market behavior in varying policy environments.

The authors deserve commendation for their effort in harmonizing complex longitudinal data across diverse national contexts, enabling a comparative analysis of labor supply responses under different welfare regimes. The scope of the research is particularly commendable, as it addresses both the extensive and intensive margins of labor market participation, enriching the discourse on gender, labor, and social policy.

While the study is methodologically robust, certain necessary assumptions warrant attention:

The use of predefined household structures may not capture the full diversity of family types, potentially limiting the generalizability of findings to less conventional or evolving family models.

Fixed income levels and standardized employment scenarios (full-time, part-time, unemployed) may oversimplify the real-life complexities and variability of employment conditions and earnings.

Assuming 100% benefit take-up ignores the real-world challenges and barriers households may face in accessing entitled benefits, possibly overstating the protective capacity of welfare systems.

Setting static percentages of average wages for income estimations may not accurately reflect the diverse economic realities faced by individuals across different sectors and regions.

While the study uses updated policy information, there may be delays in reflecting recent policy changes or economic shifts, impacting the timeliness and relevance of the analysis.

In conclusion, while the study provides significant contributions to understanding the interplay between unemployment, gender dynamics, and welfare policy, I would recommend that the authors better emphasise the limitations of their assumptions.

Reviewer #3: This is a well-done research paper, clearly adressing the question it sets out to answer: Do women increase their labour supply in response to partner's unemployment and which factors mitigate this response?

The paper strikes me as well-developed and clearly written, using good data and clearly communicating an adequate empirical strategy and can be published at this point.

The strongest aspect of this work is the careful combination of multiple datasources that were combined to clearly address each of the hypotheses.

What I consider to be the two biggest remaining weaknesses are:

1) potential anticipation of the partners unemployment, which would affect the validity of the empirical estimates: however the authors are aware of this shortcoming and honest about it in the conclusion. As this is hard to correct, I think the honest acknowledgement of this problem is the best the authors can do.

2) the unidirectional measurement of the labour market response: what i mean by this is that I suspect that there is a third unmeasured channel, and that is that if both partners are working and one of them loses employment, the female partner might become less likely to reduce her labour supply. This channel is ruled out by restricting the sample to previously unemployed or part-time working women.

As said, I consider the paper publishable at this point and comment 2) only serves as a potential avenue if the authors end up reworking the paper, as it does adequately answer the questions it set out to answer at this point.

Some minor remarks:

- a short normative discussion on advantages and disadvantages of having women increase their labour supply in response to partners income shocks would help make sense of the results a bit more (it seems like there are conflicting objectives between wanting to increasing female labour supply and making sure families are adequately insured and do not have to forego their childcare priorities etc. here). Also

- the one result where i would have a slightly different reading from the authors is with respect to childcare: they seem to read their finding that there is only an effect for mothers with children age 0-3 as showing that childcare does not have much of an effect at all, but my expectation would have been that there would probably mainly be an effect at those ages, so I am inclined to read the childcare results as being more meaningful than the authors

- a brief table with a very simple overview of the different channels (partners unemployment, interacted with marginal tax rate, interaced with childcare etc.) and the direction and strength of their effects on non-working and part-time working mothers to summarize all the results in one simple and easily place would serve the paper in terms of science communication and in terms of giving policymakers an easily readable tool of what matters for increasing female labour supply.

I recognize that the authors cannot share their data as normally requested by PLOS ONE. Providing a github with the code with which the analysis was done would be good in this case.

********** 

7. PLOS authors have the option to publish the peer review history of their article (what does this mean?). If published, this will include your full peer review and any attached files.

If you choose “no”, your identity will remain anonymous but your review may still be made public.

**Do you want your identity to be public for this peer review?** For information about this choice, including consent withdrawal, please see our Privacy Policy.

Reviewer #1: No

Reviewer #3: No

**********

[NOTE: If reviewer comments were submitted as an attachment file, they will be attached to this email and accessible via the submission site. Please log into your account, locate the manuscript record, and check for the action link "View Attachments". If this link does not appear, there are no attachment files.]

While revising your submission, please upload your figure files to the Preflight Analysis and Conversion Engine (PACE) digital diagnostic tool, https://pacev2.apexcovantage.com/. PACE helps ensure that figures meet PLOS requirements. To use PACE, you must first register as a user. Registration is free. Then, login and navigate to the UPLOAD tab, where you will find detailed instructions on how to use the tool. If you encounter any issues or have any questions when using PACE, please email PLOS at figures@plos.org. Please note that Supporting Information files do not need this step.

# Author comment: Author response to Decision Letter 0

**DOI:** 10.1371/journal.pone.0306964.r002

21 Jun 2024

We would like to thank both reviewers for their comments and suggestions. Below we have provided a point-by-point answer to each comment and suggestion received. 

Reviewer #1: This study explores the dynamic response of women's labor supply to their male partners' unemployment across 24 EU member states and the UK during the period 2009-2019. Using longitudinal data from EU-SILC and policy simulation models EUROMOD and UKMOD, the study investigates how welfare policies, particularly childcare and tax-benefit systems, influence women's decision to enter the labor market or increase working hours in response to economic shocks affecting their households.

The alleged innovation of this study lies in its comprehensive approach to examining the moderating effects of welfare policies on the labor supply decisions of women facing partners' unemployment. The research stands out for its application of harmonized data across multiple European countries and its methodological approach in using tax-benefit simulation models to analyze both the extensive (entering employment) and intensive (increasing working hours) margins of labor supply. This perspective provides a nuanced understanding of women's labor market behavior in varying policy environments.

The authors deserve commendation for their effort in harmonizing complex longitudinal data across diverse national contexts, enabling a comparative analysis of labor supply responses under different welfare regimes. The scope of the research is particularly commendable, as it addresses both the extensive and intensive margins of labor market participation, enriching the discourse on gender, labor, and social policy.

Answer: Thank you!

While the study is methodologically robust, certain necessary assumptions warrant attention:

The use of predefined household structures may not capture the full diversity of family types, potentially limiting the generalizability of findings to less conventional or evolving family models.

Answer: We fully agree that family types are highly diverse and that this study looks only at one type of families. Still such families are quite numerous in the EU (which is the region we cover), especially when children are small, and the mechanism we investigate (the added worker effect) has been widely addressed, especially in economic literature. With this study we contribute exactly to this strand of the literature by incorporating the role of the specific policies for her response to his unemployment. This is how the study was designed and preregistered after it was evaluated and accepted by several external reviewers in Plos One. However, we agree that it does not capture full diversity of family types and that the added worker affect could also be studied for same-sex couples or female breadwinner couples. Unfortunately our data would not allow us to examine it as the number of such couples in our data is too small. We admit this limitation in the paper (please see lines 70-72 and 799-803). We believe, however, our study has a potential to stimulate research for these couples on larger datasets with the use of the methodology we proposed. 

Fixed income levels and standardized employment scenarios (full-time, part-time, unemployed) may oversimplify the real-life complexities and variability of employment conditions and earnings.

Answer: Unfortunately the EU-SILC data does not allow for accounting for more nuanced employment statuses. As every dataset it has its advantages and disadvantages. The advantage is that it provides monthly retrospective data on employment for all EU member states. Its disadvantage is that the monthly calendar data provides only information on the very general employment status of the individuals (i.e. full-time employed; part-time employed, unemployed, inactive) of respondents. Consequently, using this dataset we are only able to study his unemployment and her transitions into activity and full-time employment. Having said that, we would like to mention that we have also conducted another study, which complements this one, and in which we were able to cover more diverse employment transitions (e.g. the changes in the number of her working hours in reaction to his entry into unemployment or nonstandard employment) (see Matysiak et al 2024). The study was conducted based on EU LFS data, which provided more nuanced data on employment but had other limitations (e.g. much shorter observation span than EU-SILC). All in all, we are convinced that the two studies provide a comprehensive overview of the added-worker effect in the EU and the role of the policies for moderating her response to the worsening in his employment situation which can stimulate further research on the topic. 

Assuming 100% benefit take-up ignores the real-world challenges and barriers households may face in accessing entitled benefits, possibly overstating the protective capacity of welfare systems.

Answer: We agree that assuming a 100% benefit take-up and setting static percentages of average wages constitute limitations of the study. Both limitations stem from the design of the microsimulation tool EUROMOD we employ to estimate the net replacement rates (NRR) and marginal tax rates (MRTs). As mentioned in the manuscript, by design EUROMOD assumes a 100% benefit take-up. We acknowledge this limitation in the manuscript and have stressed its implications (please see line 814-820). 

Setting static percentages of average wages for income estimations may not accurately reflect the diverse economic realities faced by individuals across different sectors and regions.

Answer: Estimating the MTRs and NRRs based on the actual income reported by the couples in our dataset is not possible. This is because the EU-SILC data as made available for research purposes is not compatible with EUROMOD. Consequently, we relied on the hypothetical household tool (HHT) provided by EUROMOD to generate hypothetical household data, which was later on used to estimate the MTRs and NRRs. In generating the hypothetical data the HHT allows users to specify wage variables as percentages of predefined country-specific average wages. These predefined wage levels cannot be adjusted so as to account for differences between regions or sectors. Moreover, the number of households generated grows exponentially with the number of categories assumed by each variable. Thus, we aimed at reducing the number of the categories to a minimum. As a result, we defined the households using static percentages of country-specific average wages. However, in order to test the sensitivity of the results we have varied the wage-thresholds used. Additionally, as already mentioned, we have acknowledged this limitation in the manuscript (please see lines 814-820). Finally, we would like to stress that this problem was raised by a reviewer of our preregistered study protocol which was accepted after our explanations. 

While the study uses updated policy information, there may be delays in reflecting recent policy changes or economic shifts, impacting the timeliness and relevance of the analysis.

Answer: We agree that there may be delays in the effects of recent policy changes and we are not able to grasp these effects with our study. However, this problem is not specific to our analysis only, but rather a common problem across all studies using large-scale survey data. Data available for the analysis always comes with a delay and empirical analyses are never able to grasp all the effects of recent policies. Furthermore, the period 2020-2022 was very specific due to the pandemic. Therefore, we decided to use the most recent data, but leave out the pandemic period. Finally, we did it exactly as planned in the (reviewed and accepted) pre-registration, which we were obliged to follow.

In conclusion, while the study provides significant contributions to understanding the interplay between unemployment, gender dynamics, and welfare policy, I would recommend that the authors better emphasise the limitations of their assumptions.

Answer: Thank you, we stressed the limitations of our assumptions in the paper as pointed out in the answers above. 

Reviewer #3: This is a well-done research paper, clearly addressing the question it sets out to answer: Do women increase their labour supply in response to partner's unemployment and which factors mitigate this response?

The paper strikes me as well-developed and clearly written, using good data and clearly communicating an adequate empirical strategy and can be published at this point.

The strongest aspect of this work is the careful combination of multiple data sources that were combined to clearly address each of the hypotheses.

Answer: Thank you!

What I consider to be the two biggest remaining weaknesses are:

1) potential anticipation of the partners unemployment, which would affect the validity of the empirical estimates: however the authors are aware of this shortcoming and honest about it in the conclusion. As this is hard to correct, I think the honest acknowledgement of this problem is the best the authors can do.

Answer: Yes, we acknowledge this problem in the conclusions.

2) the unidirectional measurement of the labour market response: what I mean by this is that I suspect that there is a third unmeasured channel, and that is that if both partners are working and one of them loses employment, the female partner might become less likely to reduce her labour supply. This channel is ruled out by restricting the sample to previously unemployed or part-time working women.

Answer: Thank you for this suggestion. Incorporating it at that stage would be against the rules of the preregistration and would substantially expand the paper. We will consider it however in our future research. 

As said, I consider the paper publishable at this point and comment 2) only serves as a potential avenue if the authors end up reworking the paper, as it does adequately answer the questions it set out to answer at this point.

Some minor remarks:

- a short normative discussion on advantages and disadvantages of having women increase their labour supply in response to partners income shocks would help make sense of the results a bit more (it seems like there are conflicting objectives between wanting to increasing female labour supply and making sure families are adequately insured and do not have to forego their childcare priorities etc. here). Also

Answer: We agree that there is a conflict between the advantages and disadvantages of an increase in women's labour supply. We have highlighted these in the section on conclusions and discussions of the manuscript (please see lines 785-797)

- the one result where i would have a slightly different reading from the authors is with respect to childcare: they seem to read their finding that there is only an effect for mothers with children age 0-3 as showing that childcare does not have much of an effect at all, but my expectation would have been that there would probably mainly be an effect at those ages, so I am inclined to read the childcare results as being more meaningful than the authors

Answer: In light of your observation we have highlighted the effect we identified for mothers with young children (please see lines 737-742). 

- a brief table with a very simple overview of the different channels (partners unemployment, interacted with marginal tax rate, interaced with childcare etc.) and the direction and strength of their effects on non-working and part-time working mothers to summarize all the results in one simple and easily place would serve the paper in terms of science communication and in terms of giving policymakers an easily readable tool of what matters for increasing female labour supply.

Answer: We have included such a table in the conclusions and discussion section of the manuscript.

I recognize that the authors cannot share their data as normally requested by PLOS ONE. Providing a github with the code with which the analysis was done would be good in this case.

Answer: The code, as well as the country-level data used in the analyses is publicly available here: https://github.com/LabFam/rEUsilience . 

References:

Matysiak, Anna, Anna Kurowska, and Alina Maria Pavelea. 2024. Adjustments in women’s labour force participation in response to the. rEUsilience Working Paper Series No 6.

Attachment Submitted filename: Answers to reviewers comments.docx

# Editor report: Decision Letter 1

**DOI:** 10.1371/journal.pone.0306964.r003

**Authors:** Jordi Gumà

26 Jun 2024

His unemployment, her response, and the moderating role of welfare policies in European countries. Results from a preregistered study.

PONE-D-24-04533R1

Dear Dr. Matysiak,

We’re pleased to inform you that your manuscript has been judged scientifically suitable for publication and will be formally accepted for publication once it meets all outstanding technical requirements.

Within one week, you’ll receive an e-mail detailing the required amendments. When these have been addressed, you’ll receive a formal acceptance letter and your manuscript will be scheduled for publication.

An invoice will be generated when your article is formally accepted. Please note, if your institution has a publishing partnership with PLOS and your article meets the relevant criteria, all or part of your publication costs will be covered. Please make sure your user information is up-to-date by logging into Editorial Manager at Editorial Manager® and clicking the ‘Update My Information' link at the top of the page. If you have any questions relating to publication charges, please contact our Author Billing department directly at authorbilling@plos.org.

If your institution or institutions have a press office, please notify them about your upcoming paper to help maximize its impact. If they’ll be preparing press materials, please inform our press team as soon as possible -- no later than 48 hours after receiving the formal acceptance. Your manuscript will remain under strict press embargo until 2 pm Eastern Time on the date of publication. For more information, please contact onepress@plos.org.

Kind regards,

Jordi Gumà, Ph.D.

Academic Editor

PLOS ONE

# Editor report: Acceptance letter

**DOI:** 10.1371/journal.pone.0306964.r004

**Authors:** Jordi Gumà

7 Jul 2024

PONE-D-24-04533R1 

PLOS ONE

Dear Dr. Matysiak, 

I'm pleased to inform you that your manuscript has been deemed suitable for publication in PLOS ONE. Congratulations! Your manuscript is now being handed over to our production team.

At this stage, our production department will prepare your paper for publication. This includes ensuring the following:

* All references, tables, and figures are properly cited

* All relevant supporting information is included in the manuscript submission,

* There are no issues that prevent the paper from being properly typeset

If revisions are needed, the production department will contact you directly to resolve them. If no revisions are needed, you will receive an email when the publication date has been set. At this time, we do not offer pre-publication proofs to authors during production of the accepted work. Please keep in mind that we are working through a large volume of accepted articles, so please give us a few weeks to review your paper and let you know the next and final steps. 

Lastly, if your institution or institutions have a press office, please let them know about your upcoming paper now to help maximize its impact. If they'll be preparing press materials, please inform our press team within the next 48 hours. Your manuscript will remain under strict press embargo until 2 pm Eastern Time on the date of publication. For more information, please contact onepress@plos.org.

If we can help with anything else, please email us at customercare@plos.org.

Thank you for submitting your work to PLOS ONE and supporting open access. 

Kind regards, 

PLOS ONE Editorial Office Staff

on behalf of

Dr. Jordi Gumà 

Academic Editor

PLOS ONE
